# Stress Triangle: Do Introduced Predators Exert Indirect Costs on Native Predators and Prey?

**DOI:** 10.1371/journal.pone.0060916

**Published:** 2013-04-09

**Authors:** Jennifer R. Anson, Chris R. Dickman, Rudy Boonstra, Tim S. Jessop

**Affiliations:** 1 School of Biological Sciences, University of Sydney, Sydney, New South Wales, Australia; 2 Centre for the Neurobiology of Stress, University of Toronto Scarborough, Ontario, Canada; 3 Department of Zoology, University of Melbourne, Melbourne, Victoria, Australia; University of Pretoria, South Africa

## Abstract

Non-consumptive effects of predators on each other and on prey populations often exceed the effects of direct predation. These effects can arise from fear responses elevating glucocorticoid (GC) hormone levels (predator stress hypothesis) or from increased vigilance that reduces foraging efficiency and body condition (predator sensitive foraging hypothesis); both responses can lead to immunosuppression and increased parasite loads. Non-consumptive effects of invasive predators have been little studied, even though their direct impacts on local species are usually greater than those of their native counterparts. To address this issue, we explored the non-consumptive effects of the invasive red fox *Vulpes vulpes* on two native species in eastern Australia: a reptilian predator, the lace monitor *Varanus varius* and a marsupial, the ringtail possum *Pseudocheirus peregrinus*. In particular, we tested predictions derived from the above two hypotheses by comparing the basal glucocorticoid levels, foraging behaviour, body condition and haemoparasite loads of both native species in areas with and without fox suppression. Lace monitors showed no GC response or differences in haemoparasite loads but were more likely to trade safety for higher food rewards, and had higher body condition, in areas of fox suppression than in areas where foxes remained abundant. In contrast, ringtails showed no physiological or behavioural differences between fox-suppressed and control areas. Predator sensitive foraging is a non-consumptive cost for lace monitors in the presence of the fox and most likely represents a response to competition. The ringtail’s lack of response to the fox potentially represents complete naiveté or strong and rapid selection to the invasive predator. We suggest evolutionary responses are often overlooked in interactions between native and introduced species, but must be incorporated if we are to understand the suite of forces that shape community assembly and function in the wake of biological invasions.

## Introduction

Increasing evidence suggests that the indirect or non-consumptive effects of predators on prey populations can often exceed the effects of direct predation [1]. In the short term such effects may include reduced access by prey to preferred habitats and food resources as well as constraints on their growth and reproduction, while longer term effects include depressed fitness and reduced abundance, site occupancy and distribution at the population level [2]–[4]. Predators may also have non-consumptive effects on each other via competition for food, especially if they are members of the same foraging guild [5].

Most research on non-consumptive interactions has focused on situations where predators have co-evolved with prey and other predators. This is not surprising; the selective pressure exerted by predators is a key determinant of the structure and function of many natural communities [6], [7]. However, the effects of introduced predators often exceed those of native predators on prey populations, dramatically reducing prey reproductive output, survival and abundance [8], [9]. These impacts appear to be wrought largely by direct predation; evidence for non-consumptive effects is limited [10], perhaps because naïve local species are often extirpated before anti-predator responses evolve [11], [12]. Despite this, recent studies predict that where local prey have large ranges or populations and survive the initial impact, a novel predator can act as a strong selective agent on prey to develop anti-predator responses [13], [14]. We test this prediction here by evaluating the non-consumptive effects of the invasive red fox (*Vulpes vulpes*) on two common native species in eastern Australia.

Two general hypotheses have been proposed to account for how predators cause non-consumptive effects [15], [16]. Firstly, the ‘predator stress hypothesis’ predicts that physiological consequences for prey of interactions with predators occur through an adrenocortical fear response [17]. This response activates the hypothalamic-pituitary-adrenal (HPA) axis and results in elevated production of glucocorticoids (GC). These hormones increase an individual’s ability to cope with a predator attack, protecting the body throughout the stress response and promoting anti-predator responses that benefit survival such as avoidance and vigilance behaviours [17]–[20]. However, anti-predator responses can be energetically costly. Persistent exposure to environmental stressors or exposure to novel stressors can result in chronic elevation of GC levels, immunosuppression, increased parasite loads and reproductive inhibition [18], [21], [22]. Predator-induced stress occurs in a range of mammal species including snowshoe hares (*Lepus americanus*) and Arctic ground squirrels (*Spermophilus parryii plesius*), with a high correlation between predation risk and plasma corticosterone levels [17], [23], [24].

Secondly, the ‘predator sensitive foraging hypothesis’ predicts that predator presence will increase prey vigilance or restrict foraging efficiency and hence result in energetic or nutritional costs [25], [26]. For example, elk (*Cervus elaphus*) in Yellowstone National Park increase their vigilance and shift from high-value foraging habitat to reduce their risk of predation by wolves (*Canis lupus*), but experience poorer nutrition as a consequence [27]–[29]. Increasing vigilance in areas dominated by raptors is beneficial also to the short term survival of partridge (*Perdix perdix*); however, the associated nutritional trade-off leads to decreased reproductive output and other long term fitness consequences [30]. Animals may show risky behaviour to obtain food if hungry [31], but increase the probability of encountering predators by doing so. The non-lethal nutritional costs of predation risk include, but are not limited to, reduced birth rate [4], [32], decline in body weight, and immunosuppression [33].

As with prey species, predators also show behavioural shifts when confronted with more dominant counterparts [34], and often suffer negative effects on body condition. For example, spotted hyena (*Crocuta crocuta*) in the Masai Mara Natural Reserve have a lower reproductive rate and food intake than in Amboseli owing to the effects of interspecific resource competition with high density lion (*Panthera leo*) populations [35]. Likewise, the red fox (*Vulpes vulpes*) is more vigilant and spends less time feeding in the presence of the larger European badger (*Meles meles*), and shows marked increases in population density when released from competition with the badger [36], [37].

The two hypotheses are not mutually exclusive; in the presence of a predator, physiological and behavioural changes can combine to reduce the fitness of prey [38] and perhaps of competing predators [39]. Indeed, in song birds, there is some evidence that these non-consumptive effects are synergistic: Clinchy *et al*. [40] showed that while reduced access to nutritional resources and increased predation pressure both result in chronic stress, the level is not additive but multiplicative.

**Figure 1:**
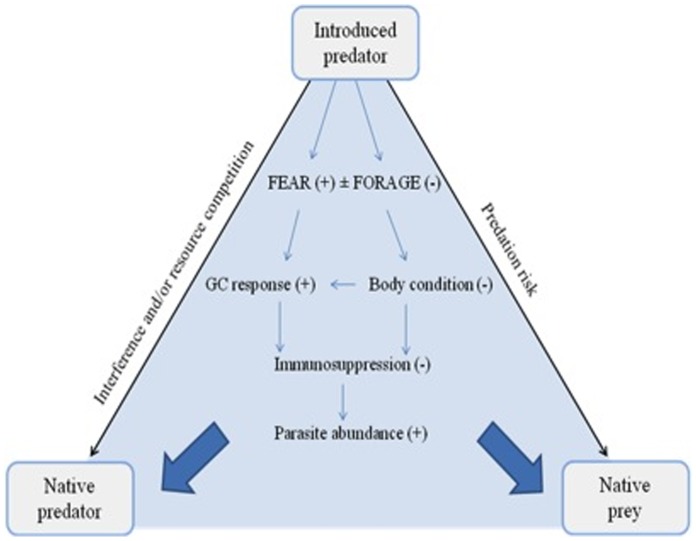
Stress triangle: Non-consumptive costs of introduced predators on native predators and prey, and the potential pathways of these costs. The ‘predator stress hypothesis’ predicts that fear will drive a chronic glucocorticoid (GC) response that leads to immunosuppression and consequent increases in parasite load. The ‘predator sensitive foraging hypothesis’ predicts that animals will reduce their foraging efficiency and hence lose body condition. This may lead directly to immunosuppression and an increase in parasite load, or indirectly by stimulating a GC response. These pathways can be set in train by an introduced predator via competition or the risk of predation on native predators and prey, respectively, forming the base of the triangle. Arrows show direction of pathway, with symbols representing an increase (+) or decrease (−) in an individual’s response.

In this study we investigate the responses of native Australian prey and predator species to the invasive red fox. We derived three predictions based on the predator stress and predator sensitive foraging hypotheses (Fig. 1), and used a landscape-scale manipulation of fox abundance to test them. Specifically, we predicted that the native species would exhibit:

1. higher plasma glucocorticoid concentrations,
2. increased vigilance and reduced risk during foraging, and
3. lower body condition and higher parasite loads

in areas where fox abundance was high compared to where it was low. We predicted that these differences would be greater in the native predator than in the prey species because, in areas with low fox abundance the native predator would be minimally exposed to the non-consumptive effects of predation but the prey would continue to be at risk from the native predator. These expectations are represented in the conceptual ‘stress triangle’ of Fig. 1. Because of mechanistic links between the two hypotheses (Fig. 1) we have not attempted to derive contrasting predictions that allow us to distinguish between the two hypotheses. However, we note that evidence for predictions 1) and 2) would support the predator stress and predator sensitive foraging hypotheses, respectively, while evidence for prediction 3) could be taken as support for both.

## Methods

### Study Species

The common ringtail possum (*Pseudocheirus peregrinus*), hereafter referred to as ringtail, and the lace monitor (*Varanus varius*) were chosen as the native study species and the red fox as the invasive predator. The fox has had pronounced effects on native species since establishment in Australia in the 1870s. It has been linked to local losses and regional extinctions of several species of vertebrates on the continental mainland, in particular small- to medium-sized mammals [41]. It affects native fauna through both predatory and competitive interactions [10].

The lace monitor is a diurnal reptile that hunts on the ground and in trees. Weighing up to 14 kg, it is the second largest native carnivore in eastern Australia [42], [43]. Its activity is seasonal, with home range varying from 185 ha in summer to zero in winter when animals are inactive [44].

The ringtail is a folivorous marsupial that feeds by night on eucalyptus leaves. Adults weigh 700–900 g and occupy home ranges of 0.02–0.05 ha [45], [46]. Animals spend some time on the ground; they utilize dreys, enclosed nest-like constructions, in addition to tree hollows [47]. The choice of dreys over hollows for nests may be a response to predation risk, as dreys more successfully facilitate escape from arboreal predators [48], [49]. Ringtails form the major prey of the fox and the lace monitor in the study region [50], [51], providing potential for competition between the two predators over this shared resource [52].

### Experimental Design

**Figure 2:**
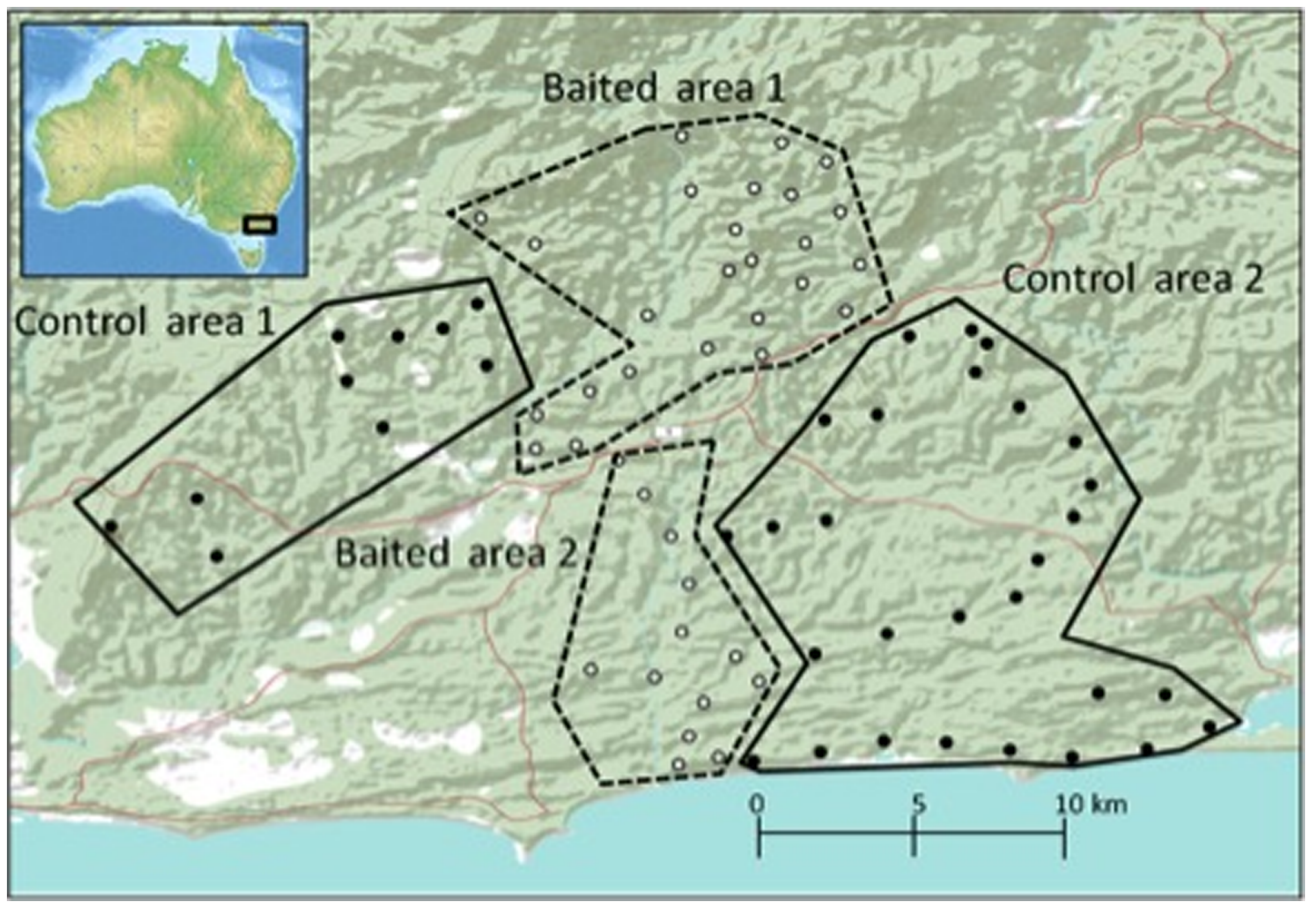
Map of the Cape Conran Coastal Park and Murrungowar state forest study region, situated in Far East Gippsland, Victoria (37°48′S 148°52′E). Dots indicate the locations of the 76 transect lines used for trap and sand pads to sample the lace monitor (*Varanus varius*). Colour of dots indicates treatment area; black represents fox (*Vulpes vulpes*) suppression areas (baited with 1080 poison) with low densities of *V. vulpes*, white represents control areas (baited with non-toxic baits) with high densities of *V. vulpes*.

The study region, covering 42,000 ha, centred on the Cape Conran Coastal Park (CCCP) and adjacent Murrungowar state forest in East Gippsland, Victoria (37°48′ S, 148°52′ E). This region was selected because its mosaic of banksia woodland, heath and lowland eucalypt forest is representative of coastal wooded habitats over much of south-eastern Australia, and also because it provides access to a regional and ongoing fox management program [53]. Initiated in 1998, this program aims to suppress fox populations and reduce their impacts on native fauna using intensive baiting with toxic ‘1080’ (sodium monofluoroacetate) baits. We used two baited ‘fox suppression’ areas and two unbaited control areas (Fig. 2), sampling predators and prey throughout to encompass the spatial variation in habitats [54], [55]. Baits are buried 15 cm below ground at ∼1 km intervals along forest management tracks in all areas; those in the fox suppression areas contain 1080 and those in the controls do not.

Independent estimates of fox activity, obtained using indexes based on baits taken and foot tracks on sand pads [56], confirm the efficacy of baiting. Whilst we acknowledge that indirect methods, such as track counts, have strengths and weaknesses with respect to their ability to explicitly detail actual population changes in fox abundance [57], they remain the most common methods for evaluating the effectiveness of poison baiting on fox population numbers. Thus we do not know how much poison baiting has reduced fox numbers, other than that fox densities in poison baited areas are considerably lower than in control areas.

Thus, fox activity declined markedly (up to a five-fold decrease in 1080 bait take) in the fox suppression areas prior to and during our study, while remaining relatively stable in the controls [53]. Lace monitors and ringtails were sampled across the study region between November 2007 and February 2010.

### Field Procedures

We captured animals to obtain blood samples and morphometric data for predictions 1 and 3, and used direct or indirect observations of foraging behaviour to test prediction 2. We captured ringtails via trapping (folding wire traps 805×265×325 mm, baited with a peanut butter, oats and rose oil mixture) and opportunistic hand capture using a noose and pole. Basal glucocorticoid blood samples were taken only from hand-captured individuals within 3 min of capture, with ∼1 ml of blood drawn from the caudal vein (27 g needle, 2 ml syringe). We collected the following measurements from all captured individuals: sex, age class, mass (g), head and pes length (mm). We assessed foraging and vigilance behaviour by measuring the heights at which animals were observed foraging and by assaying levels of responsiveness to capture. As the fox is terrestrial we assumed that ringtails foraging high above ground would experience less fox-related risk of predation than individuals lower down. Ringtails were opportunistically located by walking slowly along 500 m transects of forest tracks on dark, still nights and using a spotlight (variable beam 100 W, FaunaTech, Victoria) to detect their eye shine or movements. Using a range finder while standing under the branch or position on the tree where the ringtail was first observed, we recorded the vertical distance, or height (m), of each ringtail above the ground. Individuals were not included if movement occurred prior to the capture attempt to ensure they were not influenced by the observers. An attempt was made to capture individuals using a noose and pole, and their escape responses categorized into three vigilance levels: low (successful capture, no escape attempt), medium (delayed escape behaviour, with individuals remaining in place after initial approach), and high (immediate escape behaviour upon approach). Once an individual had been approached, the observers moved a minimum distance of 500 metres before resuming spotlighting to ensure that any individuals with overlapping home ranges were not influenced by any capture attempts.

To sample lace monitors we established 76 sites (38 in fox suppression areas, 38 in controls; Fig. 2), each containing a trap and a sand pad separated by a minimum distance of 150 m. Animals were captured using aluminium box traps (2000×300×300 mm, baited with meat and tuna emulsion oil) and by opportunistic hand capture with a noose and pole. Individuals were physically restrained using tape. We took basal glucocorticoid blood samples within 3 min of hand capture, drawing ∼3 ml of blood from the coccygeal vein (22 g needle, 5 ml syringe). We measured all individuals to obtain mass (kg), head length, head width and snout-ventral length (SVL) (cm). To assess risk-taking during foraging, we laced traps and sand pads with equal amounts of tuna emulsion oil as an attractant, but provided a greater rate of food reward (meat) in traps (350 g/day) than at sand pads (50 g/day). We assumed that animals would perceive lower foraging risks on open sand pads than at the novel structures represented by traps, and assessed the foraging choices of lace monitors as a trade-off between risk vs reward. Each site was monitored for six days during optimum weather conditions, with a mean daytime temperature above 26°C. The numbers of trap captures and sand pad detections (determined by the presence of diagnostic claw and tail marks) were tallied for each treatment to determine relative differences in foraging risk behaviour.

To avoid re-sampling, all ringtails and lace monitors were identified with a PIT tag (TROVAN® ID-100BC, Microchips, Australia, Pty Ltd.) implanted subcutaneously either between the shoulder blades (ringtails) or in the dorsal right thigh (lace monitor). Upon completion of these procedures, animals were immediately released.

### Haematology

All blood samples were stored immediately in individually heparinized containers at 4°C and prepared for analysis within 4 h of collection. Blood smears were prepared and stained using Diff-Quick (Rapid Diff, Australian Biostain Pty Ltd). Whole blood samples were then centrifuged (6000 rpm) and extracted plasma stored at −20°C until analysis. Blood smears were examined for the presence of haemoparasites at 1000× magnification under oil immersion using a compound light microscope. Five hundred erythrocytes per slide were counted and parasite load was calculated as the proportion of infected erythrocytes.

### Radioimmunoassay

We obtained glucocorticoid concentrations of total plasma cortisol in ringtails and total plasma corticosterone in lace monitors using radioimmunoassay (RIA) techniques similar to those of Jessop *et al*. [58]. Thus, plasma concentrations of cortisol were assayed using an extracted radioimmunoassay developed for foetal sheep plasma [59] with hydrocortisone (H4001, Sigma) as standard. The mean recovery of [1, 2, 6, 7 3H]-cortisol (NET396, Perkin Elmer) from ringtail plasma using dichloromethane extraction was 81.6%, with an intra-assay coefficient of variation (CV) of 16.2% and a inter-assay CV of 0.14%. Assay sensitivity was 0.33 ng/ml. The antibody had a cross reactivity of 100% with cortisol, 22% with prednisolone, 6.1% with cortexolone, 2% with cortisone, 1.3% with corticosterone and <1% with the steroids DOC and 17-hydroxy progesterone. Plasma samples of *V. varius* were extracted for corticosterone concentrations using a Corticosterone ^3^H Kit (MP Biomedicals, LLC). Final steroid concentrations were calculated from standard curves and corrected for individual sample recovery, individual plasma volume and the addition of tritiated steroid. Average (± se) sample recovery was 75.7%±0.028 with an intra-assay CV of 7.6% and an inter-assay CV of 13.04%. The antibody had 100% cross reactivity with corticosterone, 11% with 11-Dehydrocorticosterone, 7% with 11-Deoxycorticosterone, and <1% with the following steroids: progesterone, cortisol, aldosterone, testosterone, pregnenolone and 5α-DHT.

### Statistical Analysis

Statistical analyses were designed to compare the responses of the native species in areas where fox numbers were suppressed and not suppressed. All data are expressed as mean ± se, with the exception of behavioural data which are presented as percentages of the total number of individuals. Cortisol concentrations of ringtails were log_10_ transformed prior to analysis to satisfy assumptions of normality for statistical testing. Generalized linear modelling (GLM) was used to compare adrenocortical responsiveness and body condition indices for both study species. In the ringtail we derived condition indices by regressing mass on head length and in the lace monitor by regressing mass on SVL. Regression slopes were calculated using CurveExpert 1.4 (Microsoft Corporation). We also used GLM to compare foraging risk behaviour in lace monitors and sighting height and response to capture in the ringtail. Haemoparasite loads were analysed in lace monitors using a GLM Poisson log-linear model. Only one ringtail was observed to have an erythrocyte infection, so data were not analysed further. Differences were considered significant at *P*<0.05. GLM analyses were performed using SPSS (version 18) and R 2.10.1 (http://www.r-project.org).

### Ethics Statement

This study was carried out under strict accordance with animal welfare guidelines issued by the University of Melbourne. The protocols used were approved by the Animal Ethics Committee of the University of Melbourne (Permit Number: 0911328). Research was carried out on public land under Department of Sustainability Wildlife and National Parks Act (1975) research permit 10005037 and did not involve sampling of any protected species.

## Results

### Common Ringtail Possum

In total, 186 ringtails were observed and scored for height above ground when first seen; of these, 48 were captured and blood-sampled for cortisol, 150 were scored for vigilance behaviour and 56 were measured for body condition. Females and males and animals of different age were pooled for analyses after preliminary inspection using generalized linear modelling revealed no obvious differences between them in any of the response variables.

Generalized linear modelling did not detect any differences between treatments in cortisol concentrations of the ringtail (Wald χ^2^_1_ = 14.16, *P* = 0.97). After controlling for differences in the time taken to sample blood there was no elevation of glucocorticoids in response to fox predation risk. The estimated marginal means for ringtail cortisol levels were 16.77 ng/ml (±2.71) in fox suppression areas compared with 16.92 ng/ml (±2.84) in the controls.

**Figure 3:**
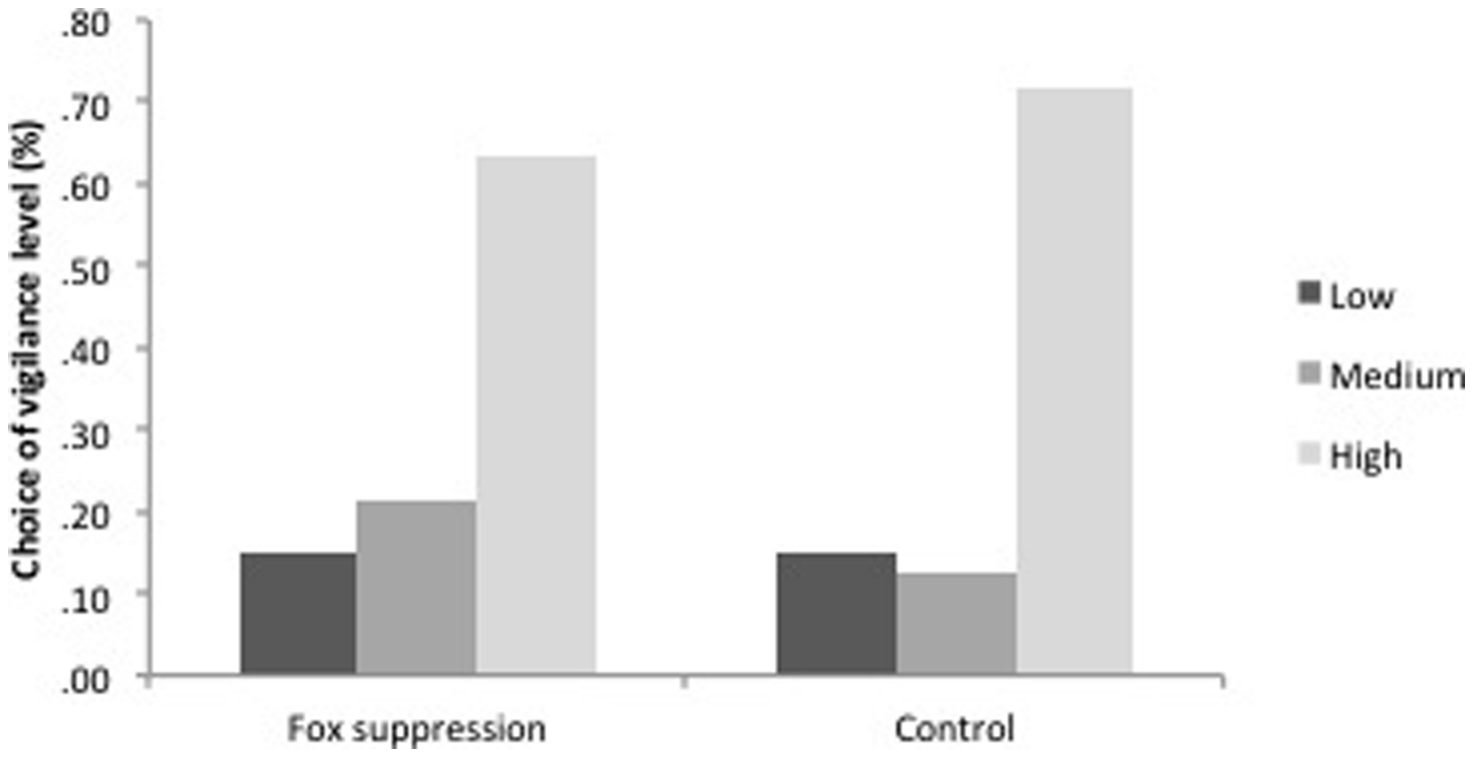
Percentage of common ringtail possums (*Pseudocheirus peregrinus*) that adopted low, medium or high vigilance behaviours in response to capture in areas of fox suppression (with low densities of foxes) and control (high densities of foxes). Capture responses were categorized into 3 vigilance levels; low (successful capture, no escape attempt), medium (delayed escape behaviour), and high (immediate escape behaviour).

No change was recorded in possum foraging behaviour, with sighting height similar between treatments (GLM: Wald χ^2^_1_ = 1.12, *P* = 0.29). Height was 5.75 m (±0.63) in the fox suppression areas compared to 4.86 m (±0.56) in the controls. Similarly, fox predation risk did not increase vigilance behaviour in ringtail, with no change in response to capture between treatments (GLM: Wald χ^2^_1_ = 0.70, *P* = 0.40) (Fig. 3).

Body condition did not differ in response to fox predation (GLM: Wald χ^2^_1_ = 1.91, *P* = 0.17). Residual values of body condition were −0.007 (±0.007) in the fox suppression areas compared to 0.006 (±0.007) in the controls. Immunosuppression was not observed through an increase in haemoparasite load; the single ringtail with an erythrocyte infection represented 3% of all animals sampled.

### Lace Monitor

In total, 45 lace monitors were observed and scored for choice of foraging strategy, and a further 21 individuals were hand captured and blood sampled for corticosterone. Of these 66 animals, 41 were assessed for haemoparasite load and 40 were measured for body condition.

There was no statistical difference in basal corticosterone levels between lace monitors in the control areas (14.97 ng/ml ±3.06) compared with those in the fox suppression areas (11.96 ng/ml ±2.55) (GLM: Wald χ^2^_1_ = 0.57, *P* = 0.45).

**Figure 4:**
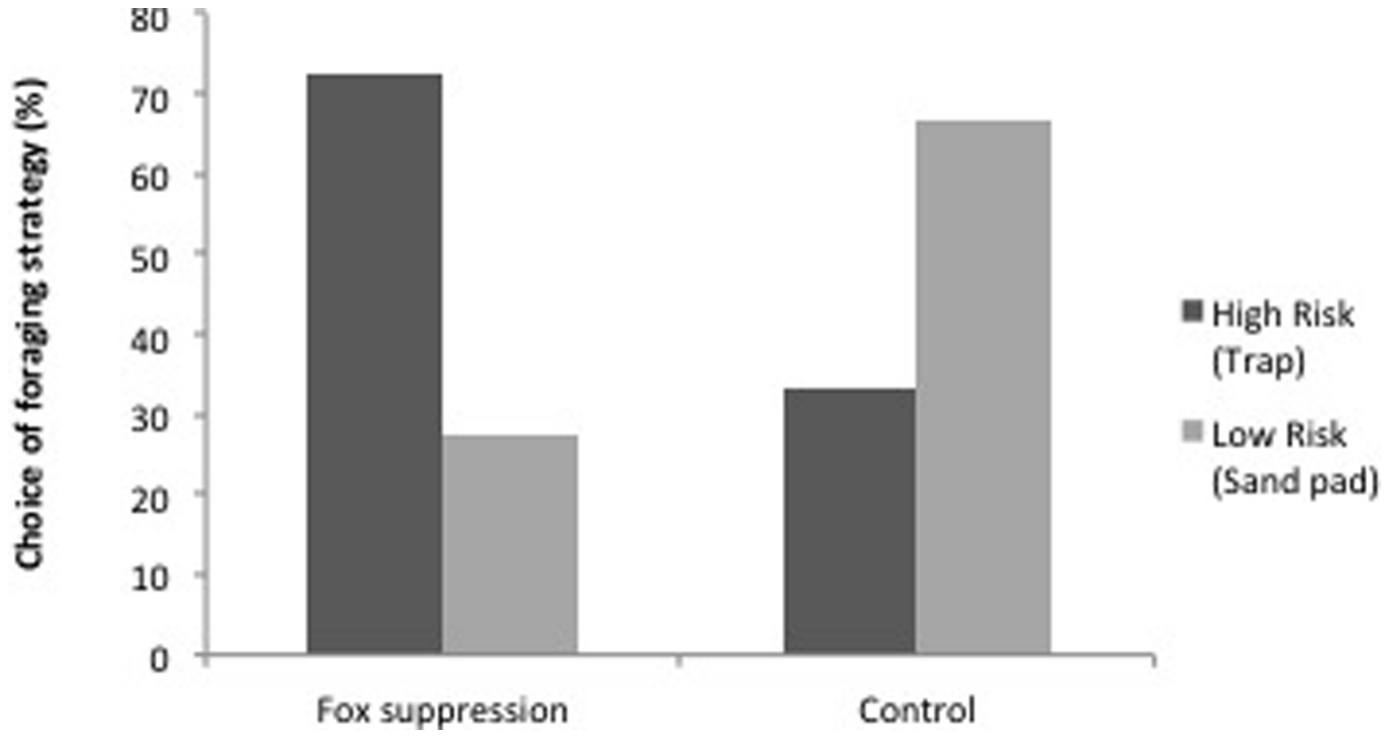
Foraging choice (%) of lace monitors (*Varanus varius*) in areas of fox suppression (with low densities of foxes) and control (high densities of foxes). Choice of foraging behaviour was between high risk-high reward sites (represented by traps) vs. low risk-low reward sites (represented by sand pads).

Foraging behaviour in lace monitors differed between the treatments (GLM: Wald χ^2^_1_ = 12.48, *P* = 0.002), representing a trade-off between risk (foraging behaviour) and reward (food). In control areas 66.7% of individuals foraged on sand pads, while 33.3% were captured. In contrast, those in fox suppression areas exhibited a shift in foraging behaviour, with 27.5% using sand pads and 72.5% being captured in traps (Fig. 4).

Body condition of lace monitors declined markedly in response to fox presence (GLM: Wald χ^2^_1_ = 5.75, *P* = 0.016). Residual values of body condition were −0.002 (±0.014) in fox suppression areas compared to −0.05 (±0.015) in the controls. The haemoparasite *Haemogregarina varanicola* was detected in 86% of lace monitor erythrocyte samples. However, fox-treatment did not affect haemoparasite load (number of parasites present in 500 red blood cells ± se); loads were 3.96±0.41 in the fox suppression areas and 3.53±0.46 in the controls (GLM: Wald χ^2^_1_ = 0.44, *P* = 0.49).

## Discussion

The results provide mixed support for our initial predictions and suggest that the native study species respond in quite different ways to the sub-lethal effects of fox presence. While neither study species showed differences in plasma glucocorticoid (GC) concentrations in areas with and without fox suppression (prediction 1), lace monitors showed changes in body condition and foraging behaviour that supported predictions 2) and 3). Ringtails, by contrast, showed no measurable response to differences in fox abundance. In the discussion below, we first review the evidence for the predator stress hypothesis and then consider the divergent behavioural and morphometric responses of the two native species to the invasive fox.

Predator-induced stress responses have been documented in a wide range of vertebrates. Acute responses typically result in sharp increases in GC levels that tail off slowly after the stressor has been removed [17], [20], [60], whereas chronic responses result in GC levels that are more persistently elevated [24]. There are several possible reasons why we failed to find any responses here. In the first instance, we potentially may have collected blood samples too slowly to detect differences in basal GC levels, instead recording levels that were rising due to the acute stress of capture. However, this is unlikely. GC concentrations typically rise within 3–5 minutes of capture in mammals [20], [61], [62], which is well within the time (three minutes) that we took to catch and bleed both lace monitors and ringtails. In addition, the GC concentrations we recorded were within the basal range reported for other vertebrate taxa [20], [60], [63]. Secondly, it is possible that our sample sizes were too low and variances so high that trends in our data were masked. Again, this seems unlikely. For the lace monitor a *post hoc* power analysis [64] suggested that a nine-fold increase in sample size would be needed to detect a between-treatment difference at α = 0.05, but for the ringtail the increase would need to exceed two orders of magnitude. Any differences in GC levels between treatments for lace monitors, and especially for ringtails, thus appear to be too slight to be meaningful. Thirdly, and most plausibly, overall stress levels were more similar between the fox suppression and control areas than we had anticipated. This interpretation is supported by the lack of haemoparasites in the ringtail and their uniform distribution between areas in the lace monitor. We explore this idea further below.

As predicted, the native predator showed the strongest response to fox presence, with improved body condition in the fox suppression areas where interspecific competition would be lowered. In other work we have shown that monitors also have lower site occupancy in areas of high fox density (unpublished data – J. Anson). Taken together, these findings suggest that foxes restrict access of the monitor to parts of the forest environment and to prey resources. As there is little evidence of intra-guild predation, this restriction most likely arises from competition for food. Of the two main types of interspecific competition interference is more common between predators, with overt agonistic behaviour often resulting in displacement of the inferior competitor [65], especially if the dominant is an invasive one [39]. In the present case, however, the diurnal activity of the lace monitor and nocturnal activity of the fox would reduce their frequency of contact and suggest that competition is at least partly exploitative in form. Temporal partitioning of activity would limit aggressive interactions, potentially attenuate stress responses, and help to explain the lack of adrenocortical elevation in the lace monitor.

Lace monitors also shifted their foraging behaviour in the way that we had predicted, avoiding risky but high reward trap sites where foxes were abundant but selecting them where foxes were suppressed. Risk-averse foraging presumably reduces access by lace monitors to food resources generally in areas where foxes remain abundant and helps to account for their poorer body condition in these areas. Given the temporal separation in the activity patterns of the two predators, however, what risks might lace monitors face from foxes? In the first instance, temporal segregation will only reduce the frequency of direct interactions but not preclude them; if there is a possibility of intense aggression or intra-guild killing [66], it would benefit lace monitors to be strongly risk-averse. Secondly, avoidance can be mediated via other channels, such as odours, even if the frequency of contact between contestants is low. Many species that fall prey to the fox or compete with it distinguish and avoid its faecal or urinary odours, thus reducing the likelihood of encountering the predator itself [14], [67], [68]; it is possible that lace monitors show similar avoidance. If this is correct, the tendency for foxes to defecate and urinate on or near traps in the study region, but much less so on sand pads [69], would probably further strengthen the perception of risk by monitors and reinforce their risk-averse behaviour in the fox control areas. Interspecific competition has been shown to promote similar changes in the foraging strategies of species that partition resources by time [70], [71]. Although we do not know how lace monitors might affect foxes, our observations suggest that monitors face reduced fitness in the presence of the fox and support other work in showing that invasive species are strong and often dominant competitors over native species [65], [72], [73].

We predicted initially that the ringtail would show weaker responses to fox manipulation than the lace monitor because it would continue to be depredated in all areas by the native predator. The absence of any obvious response was unexpected, but may be explained in several ways. Firstly, other stressors, in particular other predators, may have replaced foxes in the fox suppression areas, thus neutralizing the fox removal effect. In other studies fox suppression has led to the release of mesopredators such as the feral cat (*Felis catus*) [74] and, in the study region, has led to an increase in site occupancy of the lace monitor (J. Anson, unpublished). However, this seems an unlikely explanation for our results. On the one hand independent monitoring in the study region has shown no evident response by feral cats to the suppression of foxes [75]. On the other, if lace monitors were acting as fox ‘surrogates’ in the fox suppression areas, we would expect ringtails to show different rather than equal responses to their presence such as shifting to higher and less accessible sites in trees. There is a suite of other co-evolved predators in the study region including large forest owls (*Tyto tenebricosa*, *Ninox strenua*) and the tiger quoll (*Dasyurus maculatus*); however, the quoll is too scarce to have additional effects on ringtails and the owls are unlikely to be affected in any way by fox suppression.

A second possibility is that ringtails do not perceive foxes to be a threat and thus mount no anti-predator response. Complete naiveté is unusual as it is associated most commonly with the initial invasion phase of a new predator [12], and has been linked to local declines and extinctions of several species of Australian vertebrates [76]–[79]. Often, however, it appears that native species do respond to the risk posed by a novel predator, but the response is more appropriate to countering the threat of a coevolved predator rather than the new one [80]–[83]. Prey that employ such ineffective or energetically costly responses can be expected to experience marked consumptive impacts [12], [84]. In the present study the lack of any evident physiological or behavioural response by the ringtail means that we cannot reject the possibility that this species is naïve to the risk of fox predation. However, the lack of any consumptive effect of foxes on ringtail site occupancy and density in the study region (J. Anson, unpublished) does suggest that complete naiveté is unlikely.

Thirdly, and perhaps most intriguingly, it is possible that ringtails already have evolved effective anti-predator responses and now perceive the threat of fox predation as low and predictable. This could explain their general lack of response to fox presence, including their lack of hormonal response and negligible incidence of blood parasites. In an analogous situation, Belding’s ground squirrels (*Spermophilus beldingi*) have lower glucocorticoid levels in areas of high risk from coevolved predators, indicating that a marked hormonal response does not necessarily occur if risk is predictable or manageable [85]. There is evidence that predator recognition and avoidance can develop under strong selection on a rapid evolutionary time scale, with fox avoidance behaviours recorded in native Australian rodent species and naïve island species including Galapagos marine iguanas [14], [79], [86]. If the ringtail has been subjected to sufficiently strong selection pressure over the 130 or more years since fox introduction, the lack of obvious vigilance and anti-predator behaviours may indicate that coevolution has occurred and that ringtails now experience minimal predation risk. It is possible, for example, that the mid-level (4–6 m) foraging height of this semi-arboreal species so minimizes the risk of fox predation that the energetic costs of mounting further responses would exceed any small benefits that might accrue. As ringtails still fall prey to foxes but show no impact at the population level, it is conceivable further that only the ‘doomed surplus’ is taken [87].

Two general sets of observations are consistent with this third explanation. Firstly, early accounts of ringtail behaviour prior to the arrival of the fox suggest that it was often active on the ground [88] and was “… seldom met with in the gum-trees, [and] generally frequents the so called tea-tree scrub …” [89∶95]. As tea-tree often grows to just 3–4 m, animals using this habitat presumably would have been at some risk of predation from ground-active predators. Secondly, more recent and quantitative work shows that naïve hand-reared ringtails reintroduced to forest habitat were depredated more heavily by ground-active foxes and cats, and had a shorter survival time, than resident individuals [48]. Although these observations are not conclusive, they provide some support for the argument that the common ringtail possum has evolved effective anti-predator responses towards the fox.

If the ringtail now persists with little cost in the presence of the red fox, it may have been subject to stronger selection than has the sympatric lace monitor which still experiences non-consumptive costs from the novel predator. If correct, this could reflect greater overlap in resource use between the intra-guild competitors than between predator and prey. As ringtails now occur with foxes over most of their range the behavioural and perhaps other changes [90] that have allowed reduction in the costs of predation remain to be uncovered. However, ringtails are likely still to carry the ‘signal’ of their interaction with foxes and we predict, for example, that animals exposed to olfactory, auditory or other fox cues should be able to distinguish and avoid them compared with the cues of different predator archetypes [2], [91], [92].

While introduced predators often have direct effects on prey populations that are more strongly negative than those of native predators, our results show that they can drive non-consumptive costs as well. We suggest further that, if local species survive an invader’s initial impact and experience strong selection, these costs may be reduced rapidly to negligible levels. The evolutionary element is often overlooked in interactions between native and introduced species, but appears crucial to incorporate if we are to understand the suite of forces that shape community assembly and function in the wake of biological invasions.
